# Supplementary material for: Leveraging infectious disease models to interpret randomized controlled trials: Controlling enteric pathogen transmission through water, sanitation, and hygiene interventions
Source: PLoS Comput Biol. 2022 Dec 5;18(12):e1010748. doi: 10.1371/journal.pcbi.1010748 (PMC9754603; doi:10.1371/journal.pcbi.1010748)
Supplement: S1 Appendix — The supporting information includes the generic SISE–RCT model equations, the WASH Benefits Bangladesh SISE–RCT model equations, and supplemental results, including distributions of likelihoods, timepoint- and arm-specific relative basic reproduction numbers, and fraction of the population enrolled in the study. Fig A. Distribution of negative log-likelihood fits to the data for parameter samples in the prior and posterior sample sets. Fig B. Prevalence of self-reported diarrhea (7-day recall) in WASH Benefits comparing the baseline (red) to the combined midline (orange) and endline (yellow) surveys, as well as posterior distributions of simulated prevalence (violin plots). Fig C. Distribution of timepoint- and arm-specific relative basic reproduction numbers (R0). The dotted line corresponds to 1.00, or no difference from the control arm at baseline. The solid lines give the mean values of the distributions. Fig D. Posterior (grey) and prior (white) distributions for the estimated fraction of the population enrolled in the study. (PDF) [file pcbi.1010748.s001.pdf]

S1 Appendix: Leveraging infectious disease models to interpret randomized controlled trials: controlling enteric pathogen transmission through water, sanitation, and hygiene interventions

SISE–RCT model equations and reparameterization

The full SISE–RCT model equations for a single-intervention were given in Eq. (2) in the main text and are reproduced here:

$$\begin{aligned}
\frac{dS_+}{dt} &= -\left(\phi_{\beta_1}\beta_1 E_1 + \phi_{\beta_2}\beta_2 E_2 + \cdots + \phi_{\beta_n}\beta_n E_n\right) S_+ + \gamma I_+, \\
\frac{dI_+}{dt} &= \left(\phi_{\beta_1}\beta_1 E_1 + \phi_{\beta_2}\beta_2 E_2 + \cdots + \phi_{\beta_n}\beta_n E_n\right) S_+ - \gamma I_+, \\
\frac{dS_-}{dt} &= -\left(\beta_1 E_1 + \beta_2 E_2 + \cdots + \beta_n E_n\right) S_- + \gamma I_-, \\
\frac{dI_-}{dt} &= \left(\beta_1 E_1 + \beta_2 E_2 + \cdots + \beta_n E_n\right) S_- - \gamma I_-, \\
\frac{dE_1}{dt} &= \alpha_1(\phi_{\alpha_1} I_+ + I_-) - \xi_1 E_1, \\
\frac{dE_2}{dt} &= \alpha_2(\phi_{\alpha_2} I_+ + I_-) - \xi_2 E_2, \\
&\vdots \\
\frac{dE_n}{dt} &= \alpha_2(\phi_{\alpha_n} I_+ + I_-) - \xi_n E_n.
\end{aligned} \tag{S1}$$

The disease-free initial conditions of this system are $S_+ = \omega\rho + (1-\omega)\rho_0$ and $S_- = \omega(1-\rho) + (1-\omega)(1-\rho_0)$, with all other compartments 0.

Parameters β, α, ξ, and γ are not separately identifiable from steady-state data. We can see this fact by setting each dE_i/dt equation in Eq. S1 to 0 (a kind of quasi-steady-state assumption), solving for the E_i,

$$E_i = \frac{\alpha_i}{\xi_i}(\phi_{\alpha_i} I_+ + I_-) \tag{S2}$$

and substituting those expressions into the remaining equations.

$$\begin{aligned}
\frac{dS_+}{dt} &= -\left(\phi_{\beta_1}\frac{\beta_1\alpha_1}{\xi_1}(\phi_{\alpha_1}I_+ + I_-) + \phi_{\beta_2}\frac{\beta_2\alpha_2}{\xi_2}(\phi_{\alpha_2}I_+ + I_-) + \cdots + \phi_{\beta_n}\frac{\beta_n\alpha_n}{\xi_n}(\phi_{\alpha_n}I_+ + I_-)\right) S_+ + \gamma I_+, \\
\frac{dI_+}{dt} &= \left(\phi_{\beta_1}\frac{\beta_1\alpha_1}{\xi_1}(\phi_{\alpha_1}I_+ + I_-) + \phi_{\beta_2}\frac{\beta_2\alpha_2}{\xi_2}(\phi_{\alpha_2}I_+ + I_-) + \cdots + \phi_{\beta_n}\frac{\beta_n\alpha_n}{\xi_n}(\phi_{\alpha_n}I_+ + I_-)\right) S_+ - \gamma I_+, \\
\frac{dS_-}{dt} &= -\left(\frac{\beta_1\alpha_1}{\xi_1}(\phi_{\alpha_1}I_+ + I_-) + \frac{\beta_2\alpha_2}{\xi_2}(\phi_{\alpha_2}I_+ + I_-) + \cdots + \frac{\beta_n\alpha_n}{\xi_n}(\phi_{\alpha_n}I_+ + I_-)\right) S_- + \gamma I_-, \\
\frac{dI_-}{dt} &= \left(\frac{\beta_1\alpha_1}{\xi_1}(\phi_{\alpha_1}I_+ + I_-) + \frac{\beta_2\alpha_2}{\xi_2}(\phi_{\alpha_2}I_+ + I_-) + \cdots + \frac{\beta_n\alpha_n}{\xi_n}(\phi_{\alpha_n}I_+ + I_-)\right) S_- - \gamma I_-.
\end{aligned} \tag{S3}$$

When the resulting equations are at steady-state (i.e., setting each equation to 0), for each pathway i, the 4 pathway-specific parameters are only found in the identifiable parameter combination $\mathcal{R}_{0,i} = \frac{\beta_i\alpha_i}{\gamma_i\xi_i}$. The system of equations Eq. (4) in the main text, therefore, have the same steady state solutions as Eq. (S3).

WASH Benefits Bangladesh SISE–RCT model equations

The full and steady-state equations for the WASH Benefits Bangladesh model are as follows. For brevity, we omit the dI/dt equations, each of which is given by $dI/dt = -dS/dt$. The subscripts i on the population variables indicate the associated adherence group. Parameters β are adjusted by the time and arm-specific and relative basic reproduction numbers η_t and η_a; these propagate into the pathways-specific basic reproduction numbers in Eqs (S5). Parameters $\phi_{\alpha_w,\mathrm{S}}$ and $\phi_{\beta_f,\mathrm{H}}$ are replaced by $\bar{\phi}_{\alpha_w,\mathrm{S}}$ and $\bar{\phi}_{\beta_f,\mathrm{H}}$ in clusters without the S and H interventions, respectively, and at baseline. The full equations below are analogous to Eq. (2) in text and Eq. (S1) here.

$$
\begin{aligned}
\frac{dS}{dt} &= -S\left(\beta_w E_w + \beta_f E_f + \beta_o E_o\right) + \gamma I \\
\frac{dS_{\mathrm{W}}}{dt} &= -S_{\mathrm{W}}\left(\phi_{\beta_w,\mathrm{W}}\beta_w E_w + \beta_f E_f + \beta_o E_o\right) + \gamma I_{\mathrm{W}} \\
\frac{dS_{\mathrm{S}}}{dt} &= -S_{\mathrm{S}}\left(\beta_w E_w + \beta_f E_f + \beta_o E_o\right) + \gamma I_{\mathrm{S}} \\
\frac{dS_{\mathrm{H}}}{dt} &= -S_{\mathrm{H}}\left(\beta_w E_w + \phi_{\beta_f,\mathrm{H}}\beta_f E_f + \beta_o E_o\right) + \gamma I_{\mathrm{H}} \\
\frac{dS_{\mathrm{N}}}{dt} &= -S_{\mathrm{N}}\phi_{\beta,\mathrm{N}}\left(\beta_w E_w + \beta_f E_f + \beta_o E_o\right) + \gamma I_{\mathrm{N}} \\
\frac{dS_{\mathrm{WS}}}{dt} &= -S_{\mathrm{WS}}\left(\phi_{\beta_w,\mathrm{W}}\beta_w E_w + \beta_f E_f + \beta_o E_o\right) + \gamma I_{\mathrm{WS}} \\
\frac{dS_{\mathrm{WH}}}{dt} &= -S_{\mathrm{WH}}\left(\phi_{\beta_w,\mathrm{W}}\beta_w E_w + \phi_{\beta_f,\mathrm{H}}\beta_f E_f + \beta_o E_o\right) + \gamma I_{\mathrm{WH}} \\
\frac{dS_{\mathrm{WN}}}{dt} &= -S_{\mathrm{WN}}\phi_{\beta,\mathrm{N}}\left(\phi_{\beta_w,\mathrm{W}}\beta_w E_w + \beta_f E_f + \beta_o E_o\right) + \gamma I_{\mathrm{WN}} \\
\frac{dS_{\mathrm{SH}}}{dt} &= -S_{\mathrm{SH}}\left(\beta_w E_w + \phi_{\beta_f,\mathrm{H}}\beta_f E_f + \beta_o E_o\right) + \gamma I_{\mathrm{SH}} \\
\frac{dS_{\mathrm{SN}}}{dt} &= -S_{\mathrm{SN}}\phi_{\beta,\mathrm{N}}\left(\beta_w E_w + \beta_f E_f + \beta_o E_o\right) + \gamma I_{\mathrm{SN}} \\
\frac{dS_{\mathrm{HN}}}{dt} &= -S_{\mathrm{HN}}\phi_{\beta,\mathrm{N}}\left(\beta_w E_w + \phi_{\beta_f,\mathrm{H}}\beta_f E_f + \beta_o E_o\right) + \gamma I_{\mathrm{HN}} \\
\frac{dS_{\mathrm{WSH}}}{dt} &= -S_{\mathrm{WSH}}\left(\phi_{\beta_w,\mathrm{W}}\beta_w E_w + \phi_{\beta_f,\mathrm{H}}\beta_f E_f + \beta_o E_o\right) + \gamma I_{\mathrm{WSH}} \\
\frac{dS_{\mathrm{WSN}}}{dt} &= -S_{\mathrm{WSN}}\left(\phi_{\beta_w,\mathrm{W}}\beta_w E_w + \beta_f E_f + \beta_o E_o\right) + \gamma I_{\mathrm{WSN}} \\
\frac{dS_{\mathrm{WHN}}}{dt} &= -S_{\mathrm{WHN}}\phi_{\beta,\mathrm{N}}\left(\phi_{\beta_w,\mathrm{W}}\beta_w E_w + \phi_{\beta_f,\mathrm{H}}\beta_f E_f + \beta_o E_o\right) + \gamma I_{\mathrm{WHN}} \\
\frac{dS_{\mathrm{SHN}}}{dt} &= -S_{\mathrm{SHN}}\phi_{\beta,\mathrm{N}}\left(\beta_w E_w + \phi_{\beta_f,\mathrm{H}}\beta_f E_f + \beta_o E_o\right) + \gamma I_{\mathrm{SHN}} \\
\frac{dS_{\mathrm{WSHN}}}{dt} &= -S_{\mathrm{WSHN}}\phi_{\beta,\mathrm{N}}\left(\phi_{\beta_w,\mathrm{W}}\beta_w E_w + \phi_{\beta_f,\mathrm{H}}\beta_f E_f + \beta_o E_o\right) + \gamma I_{\mathrm{WSHN}} \\
\frac{dE_w}{dt} &= \alpha_w\left(\sum_{S\notin i} I_i + \phi_{\alpha_w,\mathrm{S}}\sum_{S\in i} I_i\right) - \xi_w E_w \\
\frac{dE_f}{dt} &= \alpha_f\left(\sum_i I_i\right) - \xi_f E_f \\
\frac{dE_o}{dt} &= \alpha_o\left(\sum_i I_i\right) - \xi_o E_o
\end{aligned}
\tag{S4}
$$

The equations for the steady states, analogous to Eq. (4) in text, do not have the same short-term dynamics of the full model but do have the same steady-state solutions. When solving these differential equations, the disease prevalence in each category in each cluster is set to 6% and the system run for 100 days.

$$
\begin{aligned}
\frac{dS}{dt} &= -S\left(\mathcal{R}_{0,w}E_w + \mathcal{R}_{0,f}E_f + \mathcal{R}_{0,o}E_o\right) + I \\
\frac{dS_{\mathrm{W}}}{dt} &= -S_{\mathrm{W}}\left(\phi_{\beta_w,\mathrm{W}}\mathcal{R}_{0,w}E_w + \mathcal{R}_{0,f}E_f + \mathcal{R}_{0,o}E_o\right) + I_{\mathrm{W}} \\
\frac{dS_{\mathrm{S}}}{dt} &= -S_{\mathrm{S}}\left(\phi_{\alpha_w,\mathrm{S}}\mathcal{R}_{0,w}E_w + \mathcal{R}_{0,f}E_f + \mathcal{R}_{0,o}E_o\right) + I_{\mathrm{S}} \\
\frac{dS_{\mathrm{H}}}{dt} &= -S_{\mathrm{H}}\left(\mathcal{R}_{0,w}E_w + \phi_{\beta_f,\mathrm{H}}\mathcal{R}_{0,f}E_f + \mathcal{R}_{0,o}E_o\right) + I_{\mathrm{H}} \\
\frac{dS_{\mathrm{N}}}{dt} &= -S_{\mathrm{N}}\phi_{\beta,\mathrm{N}}\left(\mathcal{R}_{0,w}E_w + \mathcal{R}_{0,f}E_f + \mathcal{R}_{0,o}E_o\right) + I_{\mathrm{N}} \\
\frac{dS_{\mathrm{WS}}}{dt} &= -S_{\mathrm{WS}}\left(\phi_{\beta_w,\mathrm{W}}\phi_{\alpha_w,\mathrm{S}}\mathcal{R}_{0,w}E_w + \mathcal{R}_{0,f}E_f + \mathcal{R}_{0,o}E_o\right) + I_{\mathrm{WS}} \\
\frac{dS_{\mathrm{WH}}}{dt} &= -S_{\mathrm{WH}}\left(\phi_{\beta_w,\mathrm{W}}\mathcal{R}_{0,w}E_w + \phi_{\beta_f,\mathrm{H}}\mathcal{R}_{0,f}E_f + \mathcal{R}_{0,o}E_o\right) + I_{\mathrm{WH}} \\
\frac{dS_{\mathrm{WN}}}{dt} &= -S_{\mathrm{WN}}\phi_{\beta,\mathrm{N}}\left(\phi_{\beta_w,\mathrm{W}}\mathcal{R}_{0,w}E_w + \mathcal{R}_{0,f}E_f + \mathcal{R}_{0,o}E_o\right) + I_{\mathrm{WN}} \\
\frac{dS_{\mathrm{SH}}}{dt} &= -S_{\mathrm{SH}}\left(\phi_{\alpha_w,\mathrm{S}}\mathcal{R}_{0,w}E_w + \phi_{\beta_f,\mathrm{H}}\mathcal{R}_{0,f}E_f + \mathcal{R}_{0,o}E_o\right) + I_{\mathrm{SH}} \\
\frac{dS_{\mathrm{SN}}}{dt} &= -S_{\mathrm{SN}}\phi_{\beta,\mathrm{N}}\left(\phi_{\alpha_w,\mathrm{S}}\mathcal{R}_{0,w}E_w + \mathcal{R}_{0,f}E_f + \mathcal{R}_{0,o}E_o\right) + I_{\mathrm{SN}} \\
\frac{dS_{\mathrm{HN}}}{dt} &= -S_{\mathrm{HN}}\phi_{\beta,\mathrm{N}}\left(\mathcal{R}_{0,w}E_w + \phi_{\beta_f,\mathrm{H}}\mathcal{R}_{0,f}E_f + \mathcal{R}_{0,o}E_o\right) + I_{\mathrm{HN}} \\
\frac{dS_{\mathrm{WSH}}}{dt} &= -S_{\mathrm{WSH}}\left(\phi_{\beta_w,\mathrm{W}}\phi_{\alpha_w,\mathrm{S}}\mathcal{R}_{0,w}E_w + \phi_{\beta_f,\mathrm{H}}\mathcal{R}_{0,f}E_f + \mathcal{R}_{0,o}E_o\right) + I_{\mathrm{WSH}} \\
\frac{dS_{\mathrm{WSN}}}{dt} &= -S_{\mathrm{WSN}}\left(\phi_{\beta_w,\mathrm{W}}\phi_{\alpha_w,\mathrm{S}}\mathcal{R}_{0,w}E_w + \mathcal{R}_{0,f}E_f + \mathcal{R}_{0,o}E_o\right) + I_{\mathrm{WSN}} \\
\frac{dS_{\mathrm{WHN}}}{dt} &= -S_{\mathrm{WHN}}\phi_{\beta,\mathrm{N}}\left(\phi_{\beta_w,\mathrm{W}}\mathcal{R}_{0,w}E_w + \phi_{\beta_f,\mathrm{H}}\mathcal{R}_{0,f}E_f + \mathcal{R}_{0,o}E_o\right) + I_{\mathrm{WHN}} \\
\frac{dS_{\mathrm{SHN}}}{dt} &= -S_{\mathrm{SHN}}\phi_{\beta,\mathrm{N}}\left(\phi_{\alpha_w,\mathrm{S}}\mathcal{R}_{0,w}E_w + \phi_{\beta_f,\mathrm{H}}\mathcal{R}_{0,f}E_f + \mathcal{R}_{0,o}E_o\right) + I_{\mathrm{SHN}} \\
\frac{dS_{\mathrm{WSHN}}}{dt} &= -S_{\mathrm{WSHN}}\phi_{\beta,\mathrm{N}}\left(\phi_{\beta_w,\mathrm{W}}\phi_{\alpha_w,\mathrm{S}}\mathcal{R}_{0,w}E_w + \phi_{\beta_f,\mathrm{H}}\mathcal{R}_{0,f}E_f + \mathcal{R}_{0,o}E_o\right) + I_{\mathrm{WSHN}} \\
E_w &= \sum_{S\notin i} I_i + \phi_{\alpha_w,\mathrm{S}}\sum_{S\in i} I_i \\
E_f &= \sum_i I_i \\
E_o &= \sum_i I_i
\end{aligned}
\tag{S5}
$$

Supplemental results

The negative log-likelihood distributions of the prior (parameter samples generated to uniform on their spans) and the posterior (parameter samples resampled from the prior sample set based on the negative-log likelihood) parameter samples are given in Fig A. This figure demonstrates that the resampling procedure successfully generated a posterior with an improved distribution of fits to the data.

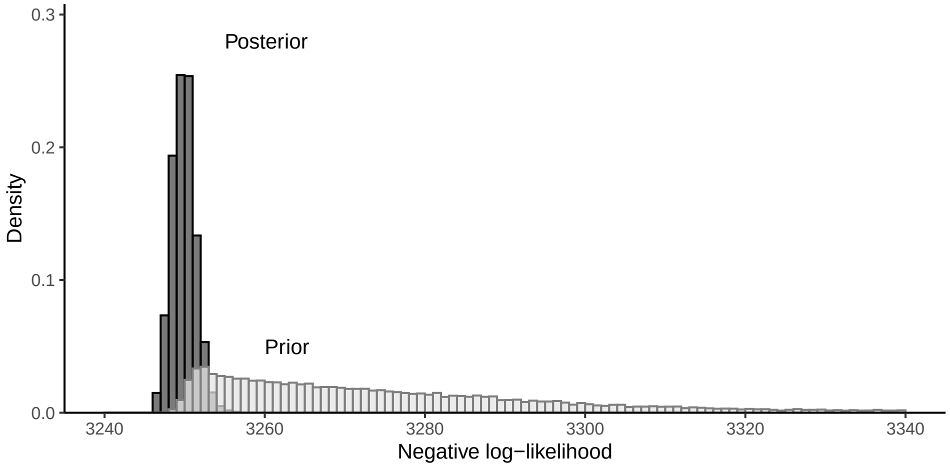

Fig A. Distribution of negative log-likelihood fits to the data for parameter samples in the prior and posterior sample sets.

A plot comparing the data by wave (not combining midline and endline) to the posterior distributions of simulated prevalence is given in Fig B.

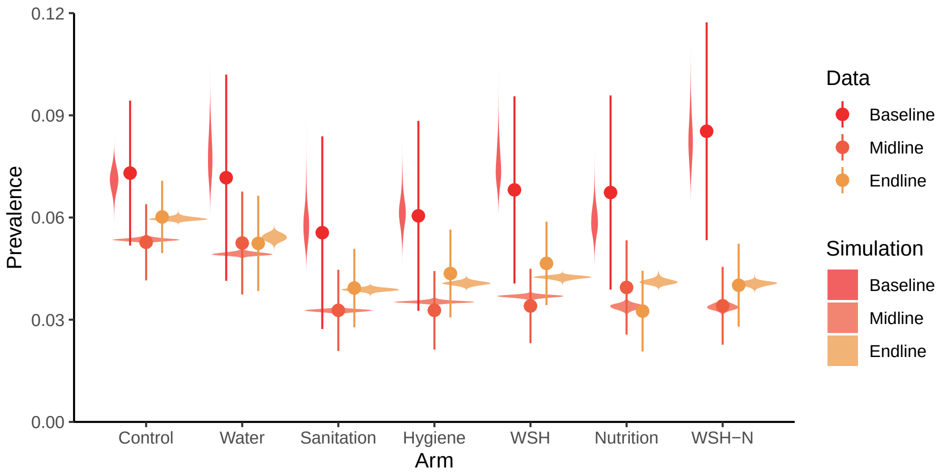

Fig B. Prevalence of self-reported diarrhea (7-day recall) in WASH Benefits comparing the baseline (red) to the combined midline (orange) and endline (yellow) surveys, as well as posterior distributions of simulated prevalence (violin plots).

The estimates of relative timepoint- and arm-specific basic reproduction numbers are given in Fig C. Both the midline and endline surveys were associated with lower reproduction numbers than the baseline survey, for all arms.

The posterior distribution of estimated fraction of the population enrolled in the study is given in Fig D.

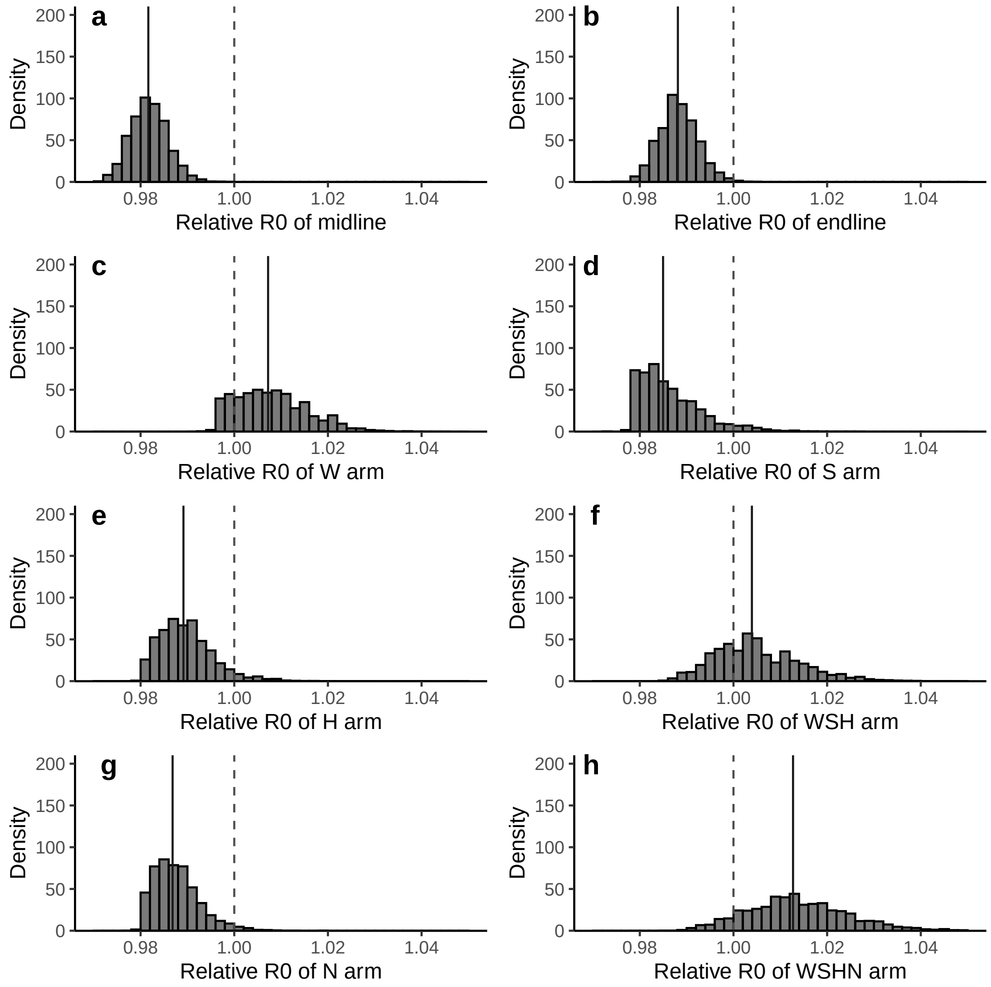

Fig C. Distribution of timepoint- and arm-specific relative basic reproduction numbers ($\mathcal{R}_0$). The dotted line corresponds to 1.00, or no difference from the control arm at baseline. The solid lines give the mean values of the distributions.

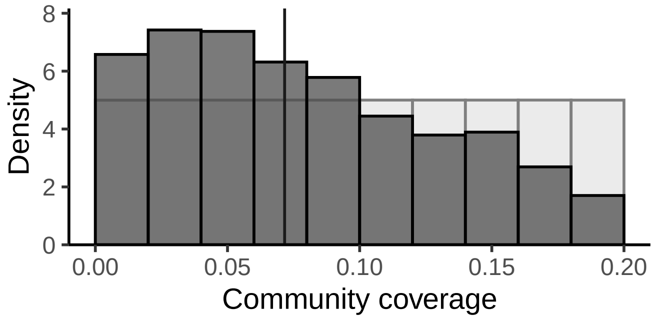

Fig D. Posterior (grey) and prior (white) distributions for the estimated fraction of the population enrolled in the study.
